# Supplementary material for: Uptake and Adherence to National Guidelines on Postpartum Haemorrhage in Italy: The MOVIE before–after Observational Study
Source: Int J Environ Res Public Health. 2023 Mar 28;20(7):5297. doi: 10.3390/ijerph20075297 (PMC10093811; doi:10.3390/ijerph20075297)
Supplement: Supplementary file 1 [file ijerph-20-05297-s001.zip › ijerph-2255845-supplementary.pdf]

Supporting information

Appendix S1. MOVIE Project – Recommendations selected from the Guideline "Postpartum haemorrhage: how to prevent it, how to treat it" – National Guideline System

Assessment of the risk factors, estimation of blood loss, early identification of critical conditions

- It is recommended to evaluate the risk factors for PPH for each woman assisted during pregnancy and at hospitalization for childbirth to identify early patients at risk
  (Recommendation of good clinical practice, based on experience of the panel)

- Given the poor accuracy of the visual estimation of blood loss, for evaluating the severity of PPH, it is recommended to consider in addition to quantification methods - graduated transparent bags, weight of gauzes and drapes, visual posters - also symptoms, clinical signs of hypovolaemia and the degree of shock.
  (Recommendation of good clinical practice, based on experience of the panel)

- It is recommended the use of graphic charts for monitoring/alerting vital signs for the early identification of critical conditions [in the post partum] since this intervention facilitates observation
  (Recommendation of good clinical practice, based on the experience of the panel)

PPH prophylaxis

- In case of vaginal birth, it is recommended to administer 10 IU IM after expulsion of the anterior shoulder or immediately after expulsion of the foetus, before clamping and cutting the funiculus for prophylaxis of PPH.
  (strong recommendation, moderate or low quality evidence)

- Oxytocin is recommended as drug of choice for the prevention of PPH in caesarean section
  strong recommendation, moderate quality evidence because indirect

Monitoring during PPH

- Arterial blood gases values, blood counts and coagulation tests ranges (PT, aPTT, fibrinogen) should be monitored regularly during bleeding
  (recommendation of good clinical practice based on the experience of the panel)

PPH pharmacological treatment and uterine massage

- In case of PPH, it is recommended as a first-line pharmacological treatment:
  - oxytocin 5 IU as a slow intravenous bolus (not less than 1-2 minutes; not less than 5 minutes in women with cardiovascular risk)
    or
  - ergometrine (2 vials 0.2 mg intramuscularly)

  or
  - association of intravenous 5 IU oxytocin (not less than 1-2 minutes; not less than 5 minutes in women with cardiovascular risk) and ergometrine (two vials 0.2 mg intramuscular) to be associated with maintenance therapy with oxytocin for infusion (10 Ul in isotonic solution for 2 hours)

strong recommendation, very low-quality evidence

- In case of PPH, it is recommended to associate the uterine massage with the pharmacological treatment until its contraction or reduction of bleeding, warning the woman that the procedure can be painful.
  strong recommendation, low quality evidence

- Early administration, within 3 hours of delivery, of 1 g of tranexamic acid slowly injected intravenously in women with PPH after vaginal delivery or caesarean section, in addition to standard treatment with uterotonics, is recommended. If bleeding persists beyond 30 minutes, or if it resumes within 24 hours from the first administration, a second dose of tranexamic acid is recommended.
  strong recommendation, high quality evidence

- When PPH is unresponsive to first-line treatment, it is recommended to consider as second-line drug treatment:
  - ergometrine (2 vials 0.2 mg intramuscular)

  and / or
  - sulprostone (1 vial 0.50 mg intravenously in 250 cc; from 0.1 to 0.4 mg / h up to a maximum of 1.5 mg in 24 hours)

  weak recommendation, very low-quality evidence

Uterine balloon

- In case of PPH unresponsive to first and second line pharmacological treatments, after excluding the presence of lacerations and rupture of the uterus or retention of placental material, the application of an intrauterine balloon before considering procedures or invasive surgery is recommended.
  (strong recommendation, low quality evidence)

Surgical procedures

- In case of failure of first and second line pharmacological treatments, promptly considering surgical procedures to stop the bleeding is recommended.
  (recommendation of good clinical practice based on the experience of the panel)

- In the case of PPH unresponsive to first and second line pharmacological treatments and to intrauterine balloon, it is recommended to consider the use of uterine compression sutures or pelvic vessel ligation or selective arterial embolization procedures based on the clinical condition of the patient, the method of delivery, the experience of health professionals and available resources.
  (strong recommendation, low quality evidence)

- It is recommended to perform hysterectomy before the patient’s condition becomes critical, especially in the case of suspected invasive abnormal placentation and/or uterus rupture or if the surgeon has no experience with conservative procedures/surgery for the treatment of severe PPH
  (strong recommendation, low quality evidence)

Transfusional therapy

- It is recommended that operative instructions on the treatment of obstetric haemorrhage in use locally contain clear indications on how to obtain emergency blood availability, including group O, RhD and K negative blood, to avoid delays in case of severe PPH.
  (recommendation of good clinical practice based on the experience of the panel)

- In case of major PPH is in progress, and if haemostasis tests are not available, it is recommended, after administering 4 units of packed red blood cells, to infuse fresh frozen plasma at doses of 15-20 mL/Kg
  (recommendation of good clinical practice based on the experience of the panel)

- When PPH is in progress it is recommended to evaluate the transfusion of platelet concentrates (1 random unit every 10 Kg of weight or equivalent from a single donor) in the presence of platelet counts below 75x109/L.
  (recommendation of good clinical practice based on the experience of the panel)

Organization of care

- It is recommended to manage the PPH with a multidisciplinary approach where the anaesthesiologist plays a crucial role in maintaining the patient's hemodynamic stability and, when necessary, in choosing and practicing the most appropriate anaesthesiological technique.
  (recommendation of good clinical practice based on the experience of the panel)

Clinical risk management

- The introduction and use of shared multidisciplinary procedures for the prevention and treatment of PPH are recommended.
  (weak recommendation, low quality evidence).

- Training and continuous multi-professional and multidisciplinary training on obstetric emergencies, including PPH, of all professionals involved in childbirth assistance are recommended
  (strong recommendation, fair quality evidence)

- Conducting periodic multi-professional simulations on the treatment of PPH is recommended.
  (weak recommendation, very low quality evidence)

- Conducting clinical audits on all cases of PPH with blood loss> 1,500 mL is recommended
  (strong recommendation, low quality evidence)

- It is recommended to ensure communication with the patient and her family members from the beginning of the bleeding
  (recommendation of good clinical practice based on the experience of the panel).

- It is recommended to offer the woman and possibly to the partner, at an appropriate time for both, a counselling on the events that characterized the PPH, specifying the risks for future pregnancies
  (recommendation of good clinical practice based on the experience of the panel).

- It is recommended that the woman is given a discharge letter containing detailed information about the EPP and any procedures / surgeries performed
  (recommendation of good clinical practice based on the experience of the panel).

Postnatal care for women with PPH and clinical risk management

- The adoption of monitoring forms for the early identification of critical conditions is recommended since this intervention facilitates observation.
  (recommendation of good clinical practice based on the experience of the panel)

Table S1. Characteristics of the Maternity Units enrolled by region in the MOVIE project

Region	Annual births in the enrolled MUs (2019)	Enrolled MUs/total MUs	Organizational model	Type of facilities
Piedmont	5,672	6/26	Hub: 3<br>Spoke: 3	Public (6)
Emilia-Romagna	6,955	6/23	Hub: 3<br>Spoke: 3	Public (6)
Tuscany	6,854	5/24	Hub: 2<br>Spoke: 3	Public (5)
Lazio	8,894	7/32	Hub: 4<br>Spoke: 3	Private with agreement (2)<br>Public (5)
Campania	11,430	9/54	Hub: 3<br>Spoke: 6	Private with agreement (3)<br>Private (1)<br>Public (5)
Sicily	9,007	10/48	Hub: 4<br>Spoke: 6	Private with agreement (2)<br>Public (8)
